# Development of population‐level colon cancer pathway concordance measures and association with survival

**DOI:** 10.1002/ijc.33964

**Published:** 2022-03-03

**Authors:** Luciano Ieraci, Maria Eberg, Katharina Forster, Paula M. Murray, Emmett Borg, Steven Habbous, Ali Vahit Esensoy, Erin Kennedy, Claire M. B. Holloway

**Affiliations:** ^1^ Data and Decision Sciences Ontario Health (Cancer Care Ontario) Toronto Ontario Canada; ^2^ Institute of Health Policy, Management and Evaluation, University of Toronto Toronto Ontario Canada; ^3^ IQVIA, 16720 Rte. Transcanadienne Kirkland Quebec Canada; ^4^ Disease Pathway Management Ontario Health (Cancer Care Ontario) Toronto Ontario Canada; ^5^ Children's Hospital Los Angeles Los Angeles California USA; ^6^ Hoffmann‐La Roche Mississauga Ontario Canada; ^7^ Quality Measurement and Evaluation Ontario Health (Cancer Care Ontario) Toronto Ontario Canada; ^8^ Klick Labs, Klick Health Toronto Ontario Canada; ^9^ Department of Surgery University of Toronto Toronto Ontario Canada

**Keywords:** colonic neoplasms, guideline adherence, medical informatics computing, pathway concordance, survival rate

## Abstract

Clinical cancer pathways help standardize healthcare delivery to optimize patient outcomes and health system costs. However, population‐level measurement of concordance between standardized pathways and actual care received is lacking. Two measures of pathway concordance were developed for a simplified colon cancer pathway map for Stage II‐III colon cancer patients in Ontario, Canada: a cumulative count of concordant events (CCCE) and the Levenshtein algorithm. Associations of concordance with patient survival were estimated using Cox proportional hazards models adjusted for patient characteristics and time‐dependent cancer‐related activities. Models were compared and the impact of including concordance scores was quantified using the likelihood ratio chi‐squared test. The ability of the measures to discriminate between survivors and decedents was compared using the C‐index. Normalized concordance scores were significantly associated with patient survival in models for cancer stage—a 10% increase in concordance for Stage II patients resulted in a CCCE score adjusted hazard ratio (aHR) of death of 0.93, 95% CI 0.88‐0.98 and a Levenshtein score aHR of 0.64, 95% CI 0.60‐0.67. A similar relationship was found for Stage III patients—a 10% increase in concordance resulted in a CCCE aHR of 0.85, 95% CI 0.81‐0.88 and a Levenshtein aHR of 0.78, 95% CI, 0.74‐0.81. Pathway concordance can be used as a tool for health systems to monitor deviations from established clinical pathways. The Levenshtein score better characterized differences between actual care and clinical pathways in a population, was more strongly associated with survival and demonstrated better patient discrimination.

## Graphical abstract

What's new?

Clinical pathways are structured, multidisciplinary plans that guide health care management for patients with specific health conditions. Here, the authors compared the actual care received by colon cancer patients with the clinical pathway maps for the disease. They developed two different measures of pathway concordance, then calculated the association between concordance and survival. This work shows that it is feasible to develop concordance measures and apply them at the population level. These measures can be used for health system monitoring and public accountability, or to assess the real‐world uptake and impact of a new intervention.

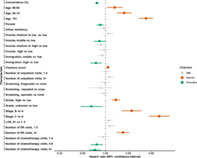

**Abbreviations**
aHR: adjusted hazard ratio
CCCE: cumulative count of concordant events
CCO: Ontario Health (Cancer Care Ontario)
cHR: crude hazard ratio
CI: confidence interval
CT: computerized tomography
df: degrees of freedom
ED: emergency department
FOBT: fecal occult blood testing
IQR: interquartile range
LOS: length of stay
MO: medical oncology
MRI: magnetic resonance imaging
OHIP: Ontario Health Insurance Plan

## 1 INTRODUCTION

Clinical pathways have been defined as structured multidisciplinary plans outlining care for patients with specific health conditions.^1^, ^2^, ^3^ With recognition of the importance of establishing and maintaining quality in increasingly complex, interconnected and technology‐driven health care systems, clinical pathways have arisen as important quality improvement tools. Their use in a variety of clinical settings has fostered improved documentation^3^, ^4^ and reduced in‐hospital complications^4^ and variation in cancer treatment.^5^ Clinical pathways can also be applied at the healthcare system level as a unifying approach to quality measurement and identification of opportunities for quality improvement. For instance, Ontario Health (Cancer Care Ontario), an agency of the government of Ontario, Canada, has developed pathway maps that provide evidence‐based recommendations about cancer management for the population, covering prevention, screening, treatment and follow‐up care delivered across diverse care settings.^6^

To realize the benefit of pathway maps, they must be implemented effectively. We refer to the extent to which actual care received by patients aligns with clinical pathway map recommendations as “pathway concordance.” A summary measure of pathway concordance could serve as a “big dot” indicator of real‐world pathway adherence for system monitoring and public accountability. It would also permit comparison of concordance across geographic areas or between specific patient groups. Analyses identifying the primary sources of discordance (eg, specific sections of the pathway or specific activities) and their relative impact on the summary measure could facilitate prioritization of actionable items for quality improvement. A summary measure could therefore inform planning, policy and program design recommendations and decisions across all phases of cancer management.

Methods of measuring pathway concordance have previously been described in published literature.^7^, ^8^, ^9^, ^10^, ^11^, ^12^, ^13^, ^14^, ^15^ However, few of these methods could be used to measure concordance with a pathway for a population. For instance, studies have compared actual care with pathway recommendations for single episodes of care (eg, an inpatient hospital stay) within single institutions^7^, ^9^, ^10^, ^13^ using data collected through clinician reporting^5^, ^8^ or manual analysis of hospital data,^11^ which are unfeasible at the population level.

Furthermore, few articles explore potential associations between pathway concordance and outcomes. Hyett et al analyzed and reported on “variances” from clinical pathways, which led to modification of certain pathways with the aim of decreasing length of inpatient stay.^8^ Konrad et al reported normalized scores for omissions and additions of healthcare events relative to clinical pathways alongside patient outcomes such as length of inpatient stay, blood glucose level and morphine use.^11^ Both higher omission scores and higher addition scores were generally associated with longer inpatient stays, but this was not statistically evaluated due to the small sample size (n = 17).

In oncology, investigating associations between cancer pathway uptake and outcomes such as patient satisfaction, survival and health system costs continues to be important.^3^ We previously measured concordance with a population‐level colon cancer pathway using a simple count of pathway‐recommended events received and found that concordance was significantly associated with 4‐year overall survival in Stage III but not Stage II colon cancer patients.^16^ Chan et al developed a summary measure of colon cancer pathway concordance that “weighted” the importance of concordance with each pathway event based on the event's impact on patient survival; this measure was also found to be associated with survival.^15^

The aim of this work was to determine the feasibility of developing and validating summary measures of pathway concordance at a population level using administrative data. We developed two measures for quantifying concordance with a colon cancer pathway: a cumulative count of concordant events (CCCE) and a variant of the Levenshtein algorithm used in information science and previously applied to stroke pathway concordance measurement.^14^ We validated both the Levenshtein algorithm and the CCCE by investigating their association with patient survival in a large cohort of colon cancer patients and compared the measures' ability to discriminate between survivors and decedents.

## 2 MATERIALS AND METHODS

### 2.1 Study setting

Ontario Health (Cancer Care Ontario), or CCO, is an agency of the government of Ontario, Canada. CCO has developed population‐level clinical pathway maps outlining best practices for the entire cancer care continuum for 11 cancers to date.^6^ Ontario's largely single‐payer healthcare system yields comprehensive administrative data from multiple institutions, presenting a unique opportunity to measure concordance of actual care with pathway maps throughout the cancer care trajectory at a population level.

CCO's pathway map for Stage II and III colon cancer was used for this work because colon cancer incidence is relatively high, the pathway map for these stages has few branch points, and significant numbers of both decedents and survivors were expected at 4 years after diagnosis, allowing comparison of concordance measures' discrimination between groups.

### 2.2 Cohort description

**FIGURE 1:**
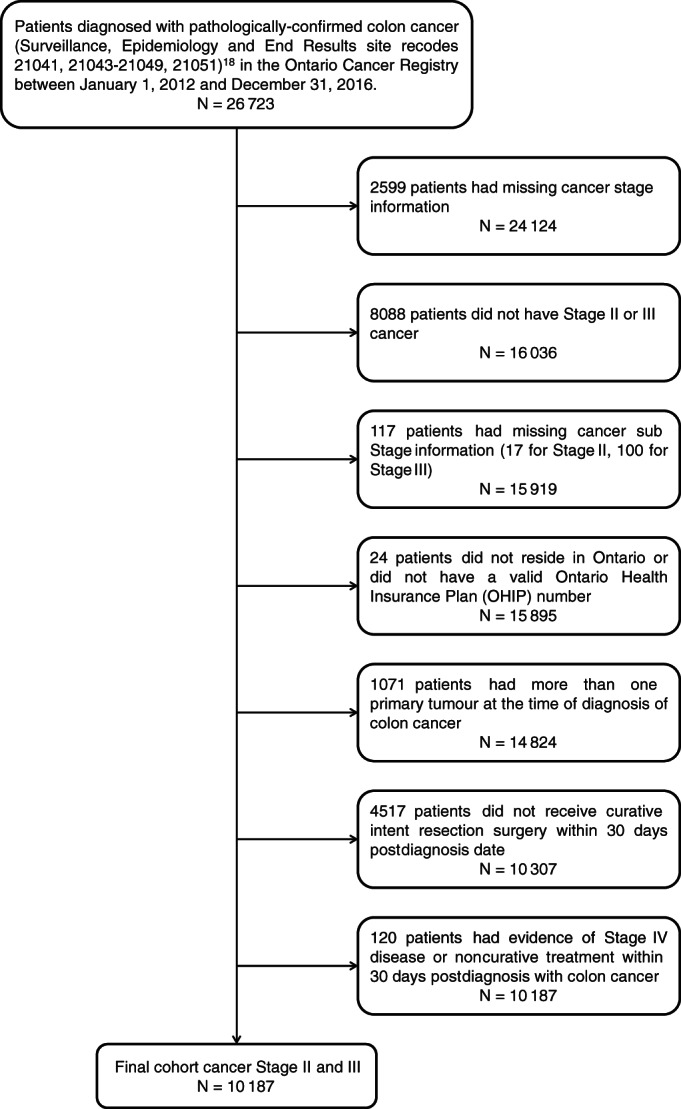
Cohort selection diagram

The study cohort included all patients diagnosed with pathologically‐confirmed Stage II and III colon cancer^17^ (Surveillance, Epidemiology and End Results site recodes 21041, 21043‐21049, 21051)^18^ in the Ontario Cancer Registry^19^ between January 1, 2012 and December 31, 2016. The American Joint Committee on Cancer has defined Stage II colon cancer as tumor stages T3‐T4b, N0, M0 and Stage III colon cancer as T1‐T4b, N1‐N2b, M0.^17^ Patients were excluded if cancer stage or substage was missing, if they did not reside in Ontario or have a valid Ontario Health Insurance Plan (OHIP) number; did not undergo resection of the primary tumor (ie, underwent treatment with noncurative intent); or had more than one primary tumor or evidence of Stage IV disease or noncurative treatment within 30 days postdiagnosis (eg, liver surgery for metastatic cancer, radiation or chemotherapy with palliative intent) (Figure 1). Follow‐up for survival analyses lasted from patients' resection date until death or censoring on March 31, 2019, whichever came first. The selection of 4 years of follow up was considered appropriate since our aim was to identify adequate numbers of survivors and decedents to compare metrics' discrimination between groups, rather than measure colon cancer survival rates for Ontario.

### 2.3 Data sources

Patients were identified across databases using OHIP health card numbers. The Activity Level Reporting database provided radiation and systemic chemotherapy data.^20^ The Ontario Drug Benefit claims database contains prescription data for patients aged 65 years or older or otherwise eligible for public coverage, and provided oral chemotherapy data.^21^ The OHIP administrative database contains information on reimbursed physician services, and provided data about clinical consultations, diagnostic imaging, diagnostic and therapeutic procedures, and screening with fecal occult blood testing (FOBT).^22^ Patients were categorized by FOBT activity using a preexisting algorithm looking back 5 years prior to diagnosis date.^23^ The National Ambulatory Care Reporting System provided emergency department (ED) visit data, chemotherapy data and Charlson Comorbidity Index Scores looking back 3 years prior to diagnosis date.^24^, ^25^, ^26^ ED visits were included to provide context about healthcare utilization patterns and potentially about patient health status. The Canadian Institute of Health Information Discharge Abstract Database contains data on admissions to acute care institutions, and provided data about surgery, inpatient chemotherapy and Charlson Comorbidity Index scores (3 year look‐back).^27^ The Registered Persons Database^28^ provided patients' postal codes of residence and death data. We used Statistics Canada's “Postal Code Conversion File Plus (PCCF+)—August 2015 Update” (version 6C) to augment the standard PCCF data using Canadian Census information on urban‐rural status, immigrant population tercile and median income quintile linked to patients' postal codes.^29^

### 2.4 Definition of reference pathways

For this feasibility study, CCO's pathway map for Stage II and III colon cancer was simplified to key events that constituted reference pathways: endoscopy (including a prior consultation with a gastroenterologist or surgeon), abdominal computerized tomography (CT) scan, pelvic CT scan, chest imaging (chest CT, magnetic resonance imaging [MRI], x‐ray or any combination thereof), primary tumor resection (including a prior consultation with a surgeon), medical oncology (MO) consultation and adjuvant chemotherapy.

Reference pathways:

1. Endoscopy + (abdominal CT + pelvic CT + [chest CT or chest x‐ray] in any order) + curative intent resection.
2. Same as (1) + MO consultation.
3. Same as (1) + MO consultation + adjuvant chemotherapy initiation + adjuvant chemotherapy completion.

CCO pathway maps recommend different care depending on patients' estimated risk of death.

**FIGURE 2:**
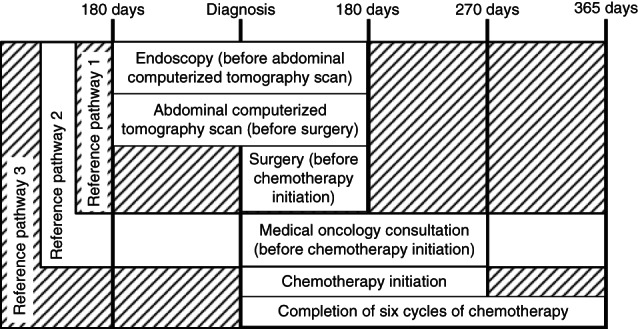
Concordant timing and sequence for reference pathway events (where required). Patients follow different reference pathways based on their estimated risk of death. Abdominal computerized tomography scan (CT) and chest imaging (chest CT scan or chest x‐ray) could occur at any point before resection. Chemotherapy initiation and completion were separated because of the differing effect on survival

Stage IIA patients could follow any of the three reference pathways. Stage IIB and IIC patients were considered to have greater risk of death, and their care was considered concordant if they followed reference pathways (2) or (3). Stage III patients' care was concordant only if they followed reference pathway (3). Pathways required some events to occur within specific time frames or sequences (Figure 2).

Data were also obtained about events that were not part of reference pathways but were included in concordance calculations: “additional” gastroenterology or surgery or any radiation oncology consultations; alternative imaging modalities (eg, chest or pelvis MRI or ultrasound, CT colonography, barium enema and small bowel x‐ray); and radiation treatment. We assumed that increased numbers of events prior to diagnosis compared to the same time period 1 year earlier were related to colon cancer symptoms or the diagnostic process. To ensure that differing concordance scores resulting from additional healthcare encounters represented clinically meaningful differences in care, we defined threshold numbers of events as concordant: up to three gastroenterology consultations or endoscopies, two abdominal CT scans and three MO consultations in the appropriate time frames were considered concordant. Healthcare events considered for generating concordance scores were observed from 180 days (6 months) prediagnosis until 365 days (1 year) postresection. The 6‐month prediagnosis interval was selected based on clinical judgment and is supported by recent research which found that 90% of Ontario colon cancer patients were diagnosed within 165 days of their first healthcare encounter related to the colorectal cancer workup.^30^

### 2.5 Pathway concordance measures

The CCCE measure was a count of observed events that matched reference pathways. For example, a Stage II patient who received events concordant with reference pathway (3) would have a CCCE value of eight: the sum of having endoscopy, abdominal CT, pelvic CT, chest CT, surgical resection, MO consultation, adjuvant chemotherapy initiation and adjuvant chemotherapy completion.

The Levenshtein algorithm was adapted from its use in information theory.^31^ Reference pathway events and observed events were represented by sequences of symbols. The algorithm determined the minimum number of symbol insertions or deletions required to make the observed event sequence match the appropriate reference pathway sequence. Insertions indicated patients were missing a reference pathway event and deletions indicated patients received additional events that were not part of the pathway. The original Levenshtein algorithm included symbol substitutions, insertions and deletions.^31^ Substitutions were not included in our study because of the difficulty interpreting the clinical relevance of “substituting” guideline‐indicated reference pathway events for each other (eg, the clinical relevance of “substituting” imaging for surgical resection compared to “substituting” MO consultation for resection).

“Scores” on the measures were defined as raw values, which were normalized and then reported as similarity percentages, where higher values indicated greater concordance. For the CCCE measure, values were normalized by dividing each patient's number of concordant events by the number of events in their longest appropriate reference pathway. For the Levenshtein measure, values were normalized by dividing each patient's number of observed insertions and deletions by the length of the observed patient pathway, or the length of the corresponding reference pathway, choosing whichever value was the greater. This value was subtracted from unity (1.0) to arrive at a concordance normalized score, where values ranged from zero (no concordant activity) to unity (all concordant activity). This corresponds to the maximum number of insertions and deletions of healthcare events possible when transforming the patient's observed pathway into the longest appropriate reference pathway.

### 2.6 Definition of variables

Patient characteristics were assigned based on their status on the date of surgical resection. Patients were categorized into FOBT screening groups using James et al's preexisting validated algorithm based on FOBT screening activity in the 5 years prior to diagnosis.^23^ Surgical resection was considered emergent if the patient was admitted through the ED. Urban (vs rural) status was assigned to patients residing in communities with greater than 10 000 persons; immigrant population terciles were based on the 2006 Census population divided nationally into three equal groups; and neighborhood income quintiles were based on 2006 household size‐adjusted income (income per single person equivalent) divided regionally by city or metropolitan area into five equal groups.^32^

### 2.7 Statistical analysis

Cohort characteristics were reported using descriptive statistics. Age was reported as a categorical variable because clinical guidelines and trial data, and therefore clinical decision‐making, as well as real‐world patient pathway assignments are typically based on age groups rather than specific years. Survival probability by stage was reported using Kaplan‐Meier plots, with “time zero” defined as the date of surgery. The relationship between the concordance measures and their ability to discriminate between living patients and decedents at the end of follow‐up were presented using density and contour plots.

Since concordance scores could change during the first year postdiagnosis, time‐dependent Cox proportional hazards models were used to estimate the association between concordance scores on both measures and death from all causes. Models were adjusted for the following covariates: patient age group (56‐64, 65‐74, 75+ years); sex; urban/rural residence; neighborhood income quintile and immigrant population tercile; number of outpatient visits in the year before cohort entry (1‐4, 5+); Charlson Comorbidity Index score; FOBT screening group^23^; cancer stage and substage; tumor grade (low, high, unknown); and postresection hospital stay longer than five days. Note Charlson Comorbidity Index was analyzed as a continuous variable in the final survival model. Covariates were assigned based on the date of surgery, or “time zero” for the survival analysis. Time‐dependent covariates included concordance score, ED visits (0, 1‐2, 3+) and chemotherapy treatments (all indications; 0, 1‐4, 5‐8, 8+ visits) occurring from resection to 1 year postresection. These time‐dependent covariates were chosen as changes in their values would signal improved survival over time (eg, improved concordance score implies patient care closer to indicated guidelines; receiving chemotherapy after resection is known to improve survival; however, an increase in ED visits is often associated with poorer patient outcomes including survival). Covariates were selected based on variables previously associated with colon cancer patient survival; variables considered potentially important confounders of pathway concordance and overall survival; clinician input; and data accessibility. Models were examined for violation of assumptions, covariate linearity and proportionality of hazards using standard tests, including visual inspection of Schoenfeld residual plots.^33^ Nonproportionality of hazards was detected for emergent presentation for cancer resection, so models were stratified on emergency surgery status to account for differing baseline hazards of death between subgroups. No other significant departure from the proportional hazards assumption was detected.

The likelihood ratio chi‐squared test for nested models was used to assess whether CCCE score added predictive value to a model with Levenshtein score and vice versa.^34^, ^35^ C‐indices measuring the models' predictive ability and discrimination were also compared.

Several sensitivity analyses were performed. This included analyses assessing the performance of the original version of the Levenshtein algorithm that incorporated character additions, deletions and substitutions and analyses excluding patients who received noncurative treatment between 30 days and 1 year postdiagnosis. Since reference pathways were derived from the CCO colon cancer pathway map for diagnosis and curative treatment, concordance for patients who received noncurative treatment was not the focus of the current analysis.

All hypothesis tests were two‐sided with statistical significance set to *P* = .05. The algorithm to calculate Levenshtein scores was implemented in Python; statistical analyses were performed using R statistical software (version 3.5.3; R Foundation).^36^

## 3 RESULTS

### 3.1 Patient characteristics and observed healthcare events

In total, 10 187 colon cancer patients were included, with median follow‐up of 3.7 years postresection (interquartile range 2.6, 3.9 years; Table 1). Almost all patients received pathway‐recommended diagnostic tests: endoscopy (88%), abdominal CT scan (98%), pelvic CT scan (98%) and chest imaging (98%). The primary source of discordance for the diagnostic phase of the pathway map was “additional,” rather than “missing,” events. Stage III patients were much more likely to receive chemotherapy than Stage II patients (70% vs 15%) and more likely to complete it (76% vs 65%).

**TABLE 1:** Description of patient cohort

Characteristic	Description	Total (n = 10 187)	Stage II (n = 4959)	Stage III (n = 5228)
Follow‐up period postresection in years, median (IQR)		3.7 (2.6, 3.9)	3.8 (2.8, 3.9)	3.5 (2.4, 3.9)
Death during observation period, n (%)		2435 (23.9%)	887 (17.9%)	1548 (29.6%)
Concordance scores, median (IQR)	Levenshtein	0.5 (0.4, 0.6)	0.5 (0.4, 0.6)	0.5 (0.4, 0.6)
	Cumulative count of concordant events	1.0 (0.8, 1.0)	1.0 (1.0, 1.0)	0.9 (0.8, 1.0)
Age group, n (%)	≤55	1541 (15.1%)	624 (12.6%)	917 (17.5%)
	56‐64	1853 (18.2%)	804 (16.2%)	1049 (20.1%)
	65‐74	2805 (27.5%)	1361 (27.4%)	1444 (27.6%)
	75+	3988 (39.1%)	2170 (43.8%)	1818 (34.8%)
Sex, n (%)	Female	4948 (48.6%)	2402 (48.4%)	2546 (48.7%)
Urban or rural residence, n (%)	Urban	8676 (85.2%)	4215 (85.0%)	4461 (85.3%)
Neighborhood income quintile, n (%)	Lowest	1991 (19.5%)	963 (19.4%)	1028 (19.7%)
	Lower‐middle	2106 (20.7%)	1020 (20.6%)	1086 (20.8%)
	Middle	1983 (19.5%)	975 (19.7%)	1008 (19.3%)
	Upper‐middle	2118 (20.8%)	1039 (21.0%)	1079 (20.6%)
	Highest	1989 (19.5%)	962 (19.4%)	1027 (19.6%)
Neighborhood immigrant population tercile, n (%)	Lowest	6362 (62.5%)	3135 (63.2%)	3227 (61.7%)
	Middle	2306 (22.6%)	1108 (22.3%)	1198 (22.9%)
	Highest	1519 (14.9%)	716 (14.4%)	803 (15.4%)
Number of outpatient visits in the year before cohort entry, median (IQR)		3.0 (1.0, 6.0)	3.0 (1.0, 6.0)	2.0 (1.0, 6.0)
Charlson Comorbidity Index score, n (%)	0	8094 (79.5%)	3878 (78.2%)	4216 (80.6%)
	1	1151 (11.3%)	602 (12.1%)	549 (10.5%)
	2+	942 (9.2%)	479 (9.7%)	463 (8.9%)
Fecal occult blood test screening group, n (%)	Repeated	335 (3.3%)	154 (3.1%)	181 (3.5%)
	Prediagnostic	1372 (13.5%)	665 (13.4%)	707 (13.5%)
	Sporadic	2897 (28.4%)	1431 (28.9%)	1466 (28.0%)
	None	5583 (54.8%)	2709 (54.6%)	2874 (55.0%)
Cancer stage subcategory, n (%)	A	4688 (46.0%)	4151 (83.7%)	537 (10.3%)
	B	3986 (39.1%)	520 (10.5%)	3466 (66.3%)
	C	1513 (14.9%)	288 (5.8%)	1225 (23.4%)
Tumor grade, n (%)	High grade	1428 (14.0%)	542 (10.9%)	886 (16.9%)
	Low grade	8410 (82.6%)	4263 (86.0%)	4147 (79.3%)
	Unknown	349 (3.4%)	154 (3.1%)	195 (3.7%)
Emergency resection, n (%)		2482 (24.4%)	1148 (23.1%)	1334 (25.5%)
Hospital length of stay (LOS) (days) postresection, median (IQR)		6.0 (4.0, 8.0)	6.0 (4.0, 8.0)	5.0 (4.0, 8.0)
Number of emergency department visits within one year postdiagnosis, n (%)	0	3020 (29.6%)	1712 (34.5%)	1308 (25.0%)
	1‐2	4402 (43.2%)	2115 (42.6%)	2287 (43.7%)
	3+	2765 (27.1%)	1132 (22.8%)	1633 (31.2%)
Number of care events during follow‐up, median (IQR)	Endoscopy	1.0 (1.0, 2.0)	1.0 (1.0, 2.0)	1.0 (1.0, 2.0)
	Abdominal CT scan	2.0 (1.0, 3.0)	2.0 (1.0, 3.0)	2.0 (1.0, 3.0)
	Pelvis CT scan	2.0 (1.0, 3.0)	2.0 (1.0, 2.0)	2.0 (1.0, 3.0)
	Chest imaging (CT or x‐ray)	3.0 (2.0, 5.0)	3.0 (2.0, 5.0)	4.0 (2.0, 5.0)
	Medical oncologist consultation	1.0 (1.0, 2.0)	1.0 (1.0, 2.0)	2.0 (1.0, 3.0)
Medical oncology consultation (one or more) within 270 days postdiagnosis and before chemotherapy initiation, n (%)		6927 (68.0%)	3253 (66.6%)	3674 (70.3%)
Any chemotherapy within 1 year postdiagnosis, n (%)		4378 (43.0%)	737 (14.9%)	3641 (69.6%)
Chemotherapy treatments within 1 year postdiagnosis, n, median (IQR)	All indications	10.0 (7.0, 12.0)	8.0 (5.0, 12.0)	11.0 (8.0, 12.0)
	Nonpalliative	9.0 (5.0, 12.0)	8.0 (3.0, 11.0)	10.0 (6.0, 12.0)
Completion of six chemotherapy cycles among patients who received any chemotherapy, n (%)		3242 (74.1%)	476 (64.6%)	2766 (76.0%)

*Notes*: Patient characteristics were assigned based on their status on the date of surgery.

Abbreviations: CT, computed tomography; IQR, interquartile range; LOS, length of stay.

### 3.2 Association between concordance and survival

**FIGURE 3:**
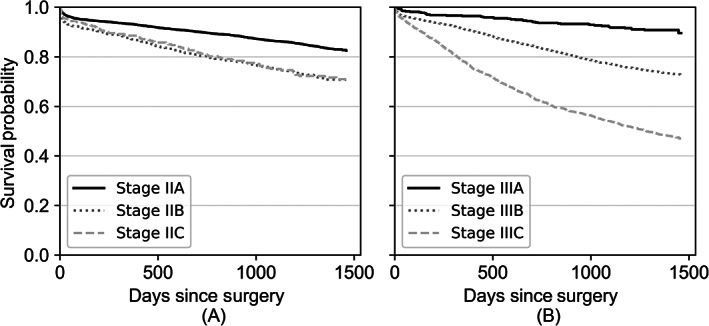
Kaplan‐Meier survival curves illustrating postsurgery survival probability for colon cancer patients stratified by cancer stage (II or III) and substage (A, B or C) at diagnosis. (A) Survival probability postsurgery for Stage II patients. (B) Survival probability postsurgery for Stage III patients

Survival estimates and Kaplan‐Meier curves were calculated for each cancer stage and substage (Figure 3). Survival rates at 4 years postresection for Stage II (Figure 3A) and III (Figure 3B) patients were 81% (95% confidence interval [CI], 79%‐82%) and 68% (95% CI, 67%‐70%), respectively. Both the CCCE and Levenshtein scores were found to have a significant association with survival in unadjusted and adjusted Cox proportional hazards survival regression. The model including Levenshtein score was reported in Table 2 due to its stronger association with survival, with the hazard ratios of significant predictors of survival summarized therein.

**TABLE 2:** Relative strength of covariate effects on likelihood of death based on Cox proportional hazards survival regression, by colon cancer stage

Covariate		Stage II			Stage III		
		aHR^a^	95% CI	*P*‐value	aHR^a^	95% CI	*P*‐value
Levenshtein concordance score (10% increase)		0.64	0.60‐0.67	<.01	0.77	0.74‐0.81	<.01
Age (years; vs ≤55)	56‐64	2.13	1.32‐3.42	<.01	1.32	1.05‐1.66	.02
	65‐74	3.15	2.03‐4.88	<.01	1.76	1.43‐2.18	<.01
	75+	8.44	5.53‐12.88	<.01	3.41	2.77‐4.19	<.01
Female (vs male)		0.79	0.69‐0.90	<.01	0.85	0.77‐0.95	<.01
Urban residence (vs rural)		0.95	0.78‐1.15	.60	0.96	0.83‐1.10	.54
Neighborhood income quintile (vs lowest)	Lower‐middle	0.91	0.75‐1.10	.33	1.09	0.94‐1.28	.25
	Middle	0.78	0.63‐0.96	.02	0.83	0.71‐0.98	.03
	Upper‐middle	0.86	0.70‐1.06	.17	0.87	0.74‐1.02	.08
	Highest	0.78	0.63‐0.98	.03	0.96	0.82‐1.13	.64
Neighborhood immigrant population tercile (vs lowest)	Middle	0.91	0.77‐1.08	.28	0.91	0.80‐1.03	.14
	Highest	0.77	0.62‐0.95	.01	0.75	0.64‐0.89	<.01
Number of outpatient visits in the year before cohort entry (vs zero)	1‐4	1.06	0.88‐1.27	.57	1.02	0.89‐1.16	.81
	5+	1.00	0.84‐1.19	.98	1.11	0.98‐1.27	.10
Charlson Comorbidity Index score (1 unit increase)		1.15	1.09‐1.20	<.001	1.08	1.03‐1.13	<.01
Fecal occult blood test screening group (vs none)	Repeated	0.74	0.46‐1.21	.23	0.87	0.62‐1.22	.42
	Prediagnostic	0.96	0.77‐1.19	.71	0.98	0.84‐1.15	.82
	Sporadic	0.99	0.84‐1.16	.86	0.96	0.85‐1.08	.46
Cancer stage subcategory (vs A)	B	1.48	1.22‐1.78	<.01	2.21	1.65‐2.96	<.01
	C	1.32	1.03‐1.69	.03	5.09	3.77‐6.87	<.01
Tumor grade (vs low)	High	1.31	1.09‐1.58	<.01	1.37	1.21‐1.54	<.01
	Unknown	0.73	0.46‐1.14	.17	0.65	0.48‐0.90	<.01
Inpatient LOS postresection >5 days (yes vs no)		1.12	0.96‐1.3	.16	1.11	0.98‐1.24	.09
Number of emergency department visits within 1 year postdiagnosis (vs zero)	1‐2	1.28	1.05‐1.57	.02	1.14	0.97‐1.34	.12
	3+	1.41	1.12‐1.78	<.01	1.69	1.42‐2.03	<.01
Number of chemotherapy treatments, all indications, within 1 year postdiagnosis (vs zero)	1‐4	1.33	0.94‐1.89	.10	0.94	0.79‐1.11	.45
	5‐8	1.23	0.84‐1.82	.29	0.77	0.64‐0.92	<.01
	8+	1.16	0.78‐1.72	.47	0.73	0.62‐0.86	<.01

*Notes*: Covariates were assigned based on their status on the date of surgery. Results were similar for models including cumulative count of concordant events score. The model including Levenshtein score was reported due to its stronger association with survival.

Abbreviations: aHR, adjusted hazard ratio; CI, confidence interval; LOS, length of stay.

^a^ aHRs were adjusted for all variables listed in this table.

Median CCCE and Levenshtein scores were significantly different for survivors and decedents (CCCE: 100% vs 80%, *P*‐value <.001; Levenshtein: 50% vs 40%, *P*‐value <.001). A 10% increase in either score was associated with improved survival in crude and adjusted models (Table 3). This association was stronger for the Levenshtein score than the CCCE score, with Stage II adjusted hazard ratio of death [aHR] 0.64 (95% CI, 0.60‐0.67) vs aHR 0.93 (95% CI, 0.88‐0.98) and Stage III aHR 0.78 (95% CI, 0.74‐0.81) vs aHR 0.85 (95% CI, 0.81‐0.88).

**TABLE 3:** Strength of estimated associations between concordance scores and death in Cox proportional hazards models, by colon cancer stage

		Stage II					Stage III				
Cohort	Concordance measure	cHR	aHR	95% CI	*P*‐value	C‐index	cHR	aHR	95% CI	*P*‐value	C‐index
Entire cohort (n = 10 187)	CCCE score (10% increase)	0.87	–	0.84‐0.91	<.01	–	0.77	–	0.75‐0.79	<.01	–
		–	0.93	0.88‐0.98	<.01	0.76	–	0.85	0.81‐0.88	<.01	0.76
	Levenshtein score (10% increase)	0.59	–	0.57‐0.62	<.01	–	0.68	–	0.66‐0.71	<.01	–
		–	0.64	0.60‐0.67	<.01	0.79	–	0.78	0.74‐0.81	<.01	0.77
Curative treatment subcohort (n = 9314)	CCCE score (10% increase)	0.87	–	0.83‐0.91	<.01	–	0.73	–	0.70‐0.75	<.01	–
		–	0.95	0.89‐1.01	.09	0.77	–	1.02	0.94‐1.10	.67	0.78
	Levenshtein score (10% increase)	0.58	–	0.55‐0.61	<.01	–	0.64	–	0.62‐0.67	<.01	–
		–	0.62	0.58‐0.65	<.01	0.80	–	0.74	0.70‐0.78	<.01	0.79

*Notes*: See Section 2 for description of model covariates.

Abbreviations: aHR, adjusted hazard ratio; CCCE, cumulative count of concordant events; cHR, crude hazard ratio; CI, confidence interval.

### 3.3 Comparison of concordance measures

**FIGURE 4:**
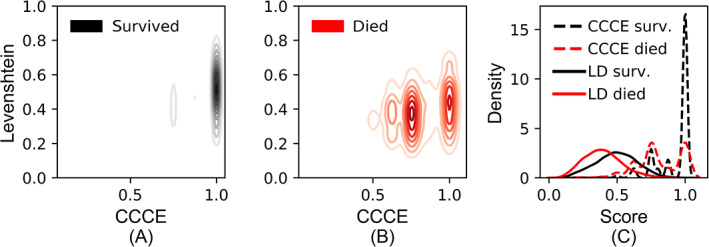
Comparison of discrimination performance of the Levenshtein and cumulative count of concordant events (CCCE) measures. (A) Contour plot illustrating frequency of concordance scores on both measures showing the measures' discrimination between patients who survived. (B) Similar contour plot to (A) showing discrimination between decedents. (C) Probability density plot illustrating the distribution of concordance scores on both measures and discrimination between survivors and decedents [Color figure can be viewed at wileyonlinelibrary.com]

The Levenshtein measure resulted in a wider range of scores which discriminated between survivors and decedents better than CCCE scores (Figure 4). Most patients had a CCCE score of 100% concordance, including 70% of patients who survived, while their Levenshtein scores ranged from 20% to 80% (Figure 4A). Among decedents (Figure 4B), CCCE scores ranged from 50% to 100%, with most scores concentrated between 63% and 75%, while Levenshtein scores ranged from 10% to 80%. A probability density plot is shown in Figure 4C, which illustrates how the CCCE and Levenshtein scores compare—both distributions show higher scores among patients who survived.

Sources of discordance contributing to lower Levenshtein scores for individual patients were more often additional events (observed in 99% of patients with discordance) than missing events (observed in 57% of patients with discordance). However, many Stage III patients did not have concordant chemotherapy activities: 30% did not initiate chemotherapy and 24% did not complete it. Of the Stage III patients with CCCE scores below 100%, indicating “missing” activity, 72% were “missing” initiation of chemotherapy and 18% initiated chemotherapy but were “missing” completion.

Nested model testing^34^ determined that the Levenshtein score added significant predictive value to a survival model including CCCE score (Stage II: *χ* ^2^ = 242.73, degrees of freedom [df] = 1, n = 4959, *P*‐value <.001; Stage III: *χ* ^2^ = 97.42, df = 1, n = 5228, *P*‐value <.001). Adding the CCCE score to a model including Levenshtein score only added predictive value for Stage III patients (Stage II: *χ* ^2^ = 0.0729, df = 1, n = 4959, *P*‐value = .7872, Stage III: *χ* ^2^ = 38.59, df = 1, n = 5228, *P*‐value <.001). Survival models adjusted for Levenshtein score had greater discrimination, though this was only significant for Stage II patients (C‐index difference: Stage II *P*‐value <.001; Stage III *P*‐value = .1779).

In a sensitivity analysis, 873 patients (8.6%) who received noncurative treatment within 1 year postdiagnosis were excluded. Among the remaining curative treatment subcohort (n = 9314), 82% of Stage II patients (95% CI, 80%‐83%) and 72% of Stage III patients (95% CI, 71%‐73%) were alive at the end of follow‐up. In adjusted multivariable analysis, Levenshtein score, but not CCCE score, was independently associated with better survival (Table 3). The Levenshtein score also added significant predictive value to models with CCCE for Stage II and III patients (Stage II: *χ* ^2^ = 254.67, df = 1, n = 4754, *P*‐value <.001; Stage III: *χ* ^2^ = 132.65, df = 1, n = 4560, *P*‐value <.001), whereas adjustment for CCCE score did not improve prediction for models with Levenshtein score (Stage II: *χ* ^2^ = 0.2595, df = 1, n = 4754, *P*‐value = .6104; Stage III: *χ* ^2^ = 1.609, df = 1, n = 4560, *P*‐value = .2046).

Another sensitivity analysis found that using the original version of the Levenshtein algorithm that incorporated additions, deletions and substitutions of characters representing healthcare events did not generate considerably different scores from those reported here for the Levenshtein algorithm reflecting only additions and deletions. More specifically, survival analysis results were found to be similar to the current analysis, including both the significance of covariates and the magnitude of estimated effects. The results of the latter Levenshtein algorithm's scores, reflecting “additional” or “missing” pathway events, were believed to be more clinically meaningful and more easily actionable.

## 4 DISCUSSION

Our work demonstrates that it is feasible to develop valid summary pathway concordance measures that are derived from administrative data and can be applied to the entire cancer care trajectory at the population level. Both CCCE and Levenshtein measures were independently associated with survival; however, Levenshtein score incorporated both “additional” and “missing” events, was more predictive of survival, and showed superior patient discrimination. When patients receiving noncurative treatment were excluded, Levenshtein score added predictive value to models adjusted for CCCE score for both Stage II and III patients. Reflecting “additional” events is an important feature for a concordance measure, as almost all patients with Levenshtein scores of less than 100% concordance had “additional” events. “Additional” events were also the primary source of discordance for the diagnostic segment of the pathway map, which is consistent with our previous findings in a different cohort.^16^ Thus, the Levenshtein score may be more useful for performance management compared to simpler count measures like the CCCE. However, the CCCE may be more easily interpreted by end users of concordance measures such as clinicians and healthcare administrators. Further evaluation of both metrics with end users and other stakeholders could inform decisions about which metric to use in different contexts.

Our results somewhat contrast those of Williams et al, who tested various methods of measuring pathway concordance and found that a version of the Levenshtein algorithm incorporating substitution or switching of care events best discriminated between stroke patients.^14^ However, their study population was limited to a single unit in a single institution and concordance was measured over the finite duration of an inpatient stay; commonly switched pathway events were “arrive stroke bed” and “brain scan.” CCO's population‐level pathways include more types of healthcare events occurring over a longer time, making substitution of events less likely and making it difficult to interpret the clinical implications of substitution. As we are interested in using the algorithm for health system monitoring, identifying sources of discordance among different subsets of patients, and identifying health system quality improvement opportunities, it is more important for us to capture “additional” or “missing” pathway activities as opposed to “substituted” activities, so substitutions were not included in the Levenshtein calculation. Note that an algorithmic substitution can be rewritten as a pathway activity deletion and insertion, so no loss in generality arises due to our omission of substitutions, and the definitions of concordant events in the reference pathways included the appropriate sequences for events. Furthermore, in separate sensitivity analyses, including substitutions when calculating the Levenshtein score showed highly similar associations with patient survival, supporting our decision to adopt the simpler version of the score calculated without substitutions.

There are many potential applications of population‐based pathway concordance measures. They may serve as “big dot” indicators for health system monitoring and public accountability, or be used to model and assess the real‐world uptake and impact of a new intervention (eg, new guideline, diagnostic test, treatment) on downstream pathway elements and patient and system outcomes (such as cost). Concordance may be measured across the entire cancer care trajectory, or segments of it, depending on the requirements of the end user. Unlike measures which quantify adherence to a single pathway map element, the summary measure facilitates detection of variation in integration or coordination of care between one or more elements. Identification of the pathway elements contributing most to discordance can highlight potential opportunities for quality improvement. Concordance measures that rely on administrative data may be applied to the population of an entire health region, as in our study, or a single institution or catchment area. In this way, a unified approach to pathway concordance measurement may be leveraged for multiple measurement applications throughout the health care system. To improve accuracy and utility, we would propose that concordance be measured against the full, rather than simplified CCO colon cancer pathway map prior to implementation of the measure for system monitoring.

This feasibility study does not explain the basis for the independent association of pathway concordance with survival; lower concordance does not imply substandard or unnecessary clinical care. For instance, chemotherapy is associated with improved survival for Stage III colon cancer patients; however, it was the most common “missing” event contributing to reduced concordance scores. This reduced concordance may reflect patient choice and other unmeasured contraindications to chemotherapy^16^, ^37^, ^38^ or patient death before chemotherapy initiation rather than substandard care. Similarly, additional events may reflect comorbidities, complications or symptoms which themselves may be associated with reduced survival, indicating that pathway concordance for individual patients may be explained by other factors unrelated to substandard clinical care. Thus, reduced concordance observed among some patient subcohorts is hypothesis‐generating.

There are several limitations to our study. First, with a relatively low maximum value of 8, the CCCE score's resolution and discrimination between patients was inherently limited. Nevertheless, the CCCE was explored because its simplicity may facilitate interpretation by end users of concordance measures, making it preferable under certain circumstances. Second, oral chemotherapy data are unavailable for patients under 65 and ineligible for publicly funded drugs, so the association of concordance with survival may have been underestimated for these patients. Given the older cohort (67% were 65+ years of age) and tendency to prescribe intravenous chemotherapy for younger colon cancer patients,^37^, ^39^ the impact of these missing data is likely small. Third, the use of simplified reference pathways in our study classified some appropriate encounters and interventions as “additional.” Fourth, as our concordance measures do not include event timing in their calculations, we defined appropriate time frames and sequences for concordant pathway map events based on previous research and clinical judgment. However, our concordant event definitions and lookback periods may not capture all pathway‐related activities for all patients. Concordance measures could be further refined by incorporating the actual time between events. There were also some data‐related limitations. We calculated Charlson comorbidity scores using inpatient and ambulatory hospital encounters but not patient encounters in other health settings, which may underrepresent the true comorbidity burden in the population. Finally, the concordance measures gave all reference pathway events equal weight, implying that they are equally important to survival and clinical decision‐making. Data‐dependent and data‐independent methods of estimating weights for each reference pathway event are being investigated by the authors to improve measure performance.

## 5 CONCLUSION

Our study demonstrated the feasibility of developing summary population‐level pathway concordance measures with scores associated with survival that could be used to assess healthcare system performance, identify quality improvement opportunities and model the effect of new interventions on downstream pathway map elements and outcomes. The Levenshtein measure accounted for more types of deviation from reference pathways and was more predictive of survival. Additional studies are required to refine the accuracy and face validity of the Levenshtein measure and explore its applicability to other disease sites and outcomes of interest.

## CONFLICT OF INTEREST

None.

## ETHICS STATEMENT

Ontario Health (Cancer Care Ontario) is designated a “prescribed entity” for the purposes of section 45(1) of the Personal Health Information Protection Act of 2004. As a prescribed entity, Ontario Health (Cancer Care Ontario) is authorized to collect personal health information from health information custodians without the consent of the patient, and to use such personal health information for the purpose of analysis or compiling statistical information with respect to the management, evaluation or monitoring of the allocation of resources to or planning for all or part of the health system, including the delivery of services. Because our study was conducted in support of OH (CCO)'s mandate for health system monitoring and planning, and is in compliance with privacy regulations, ethics review was not required.

## Data Availability

The data that support the findings of our study are available from Ontario Health (Cancer Care Ontario). Further details are available from the corresponding author upon request.
